# Enzymatic Synthesis of Maltitol and Its Inhibitory Effect on the Growth of *Streptococcus mutans* DMST 18777

**DOI:** 10.3390/biom12020167

**Published:** 2022-01-20

**Authors:** Patinya Haewpetch, Prakarn Rudeekulthamrong, Jarunee Kaulpiboon

**Affiliations:** 1 Protein Research Laboratory, Department of Pre-Clinical Science, Division of Biochemistry, Faculty of Medicine, Thammasat University, Pathumthani 12120, Thailand; patinya.haew@dome.tu.ac.th; 2 Department of Biochemistry, Phramongkutklao Hospital, Phramongkutklao College of Medicine, Bangkok 10400, Thailand; prakarn_r@pcm.ac.th

**Keywords:** antibacterial activity, anticariogenic activity, cyclodextrin glycosyltransferase (CGTase), maltitol, *Streptococcus mutans*

## Abstract

This study aimed to synthesize maltitol using recombinant CGTase from *Bacillus circulans* A11 with β-cyclodextrin (β-CD) and sorbitol as a glucosyl donor and acceptor, respectively, and assess its antibacterial activity. Optimal conditions for producing the highest yield, 25.0% (*w*/*w*), were incubation of 1% (*w*/*v*) β-CD and sorbitol with 400 U/mL of CGTase in 20 mM phosphate buffer at pH 6.0 and 50 °C for 72 h. Subsequently, maltitol underwent large-scale production and was purified by HPLC. By mass spectrometry, the molecular weight of the synthesized maltitol was 379.08 daltons, corresponding exactly to that of standard maltitol. The relative sweetness of synthesized and standard maltitol was ~90% of that of sucrose. Spot assay on the agar plate showed that maltitol inhibited the growth of *Streptococcus mutans* DMST 18777 cells. In addition, the MIC and MBC values of synthesized and standard maltitol against *S. mutans* were also determined as 20 and 40 mg/mL, respectively. These results show that the synthesized maltitol can be produced at high yields and has the potential to be used as an anticariogenic agent in products such as toothpaste.

## 1. Introduction

Enzymatic transglycosylation has been utilized in food and medicinal chemistry to synthesize oligosaccharides, polysaccharides and saccharide derivatives, which can lead to changes in their physiological and chemical properties such as water solubility and sensory characteristics such as mouth-feel taste (sweetness, bitterness and astringency) [1,2]. Cyclodextrin glycosyltransferase (CGTase—E.C. 2.4.1.19), a glycosidase, is an enzyme that catalyzes the transglycosylation reaction, which transfers the α-1,4 glucan from a glycosyl donor to another glucan acceptor such as starch, related oligo/polysaccharides, and sugar alcohols with a free 4-hydroxyl group [3]. The transglycosylation of CGTase consists of intramolecular and intermolecular transglycosylation reactions. Intramolecular transglycosylation (cyclization reaction) results in the formation of cyclic oligosaccharides, e.g., cyclodextrin (CD) and intermolecular transglycosylation can occur through three different reactions. The major reaction is disproportionation, in which a glycosyl is transferred from a donor to an acceptor oligo/polysaccharide or sugar alcohol with a free 4-hydroxyl group resulting in a linear oligo/polysaccharide or sugar alcohol of variable length. The second reaction is coupling, a reversal of cyclization, and the third, hydrolysis, is a low activity reaction with water acting as a glycosyl acceptor [4,5,6,7,8].

Kim et al. [7] synthesized glucosyl-maltitol using CGTase with starch as the glucosyl donor and maltitol as the glucosyl acceptor. The obtained transglycosylation products were purified and their chemical structures identified. They also reported the synthesis of transglucosylated xylitol using *Thermoanaerobacter* sp. CGTase and their bifidogenic effects [8]. Interestingly, they found that maltosyl-xylitol promoted the growth of *Bifidobacterium breve* compared to original xylitol, indicating the possibility of a new alternative sweetener that could be used as a prebiotic. Moreover, sugar alcohols are known to be less reactive than the corresponding sugars due to the absence of reducing carbonyl groups and the presence of oxygen atoms instead of hydroxyl groups [9]. Hence, they are the alternative to sugar as a sweetener because of their low caloric value and glycemic index, which prevent hyperglycemia in diabetes and dental caries [10,11]. Sugar alcohols are also used in dietetic food as humectants to control water activity and moisture in foods [6].

Presently, dental caries is one of the most critical health concerns for humans of all ages, especially in childhood, where the number of children with dental caries continues to rise [12]. Dental caries is mainly promoted by organic acids such as lactic acid from cariogenic bacteria. The organic acids produced create an acidic environment that induces demineralization of tooth enamel [13]. Of the cariogenic bacteria, *Streptococcus mutans* is the main microorganism that causes dental caries because of its ability to ferment lactic acid [14]. Fluoride has been widely used as an anticariogenic substance and forms a complex with magnesium and enolase in the glycolysis pathway. This complex decreases the phosphoenolpyruvate (PEP) level of *S. mutans* cells, resulting in a lack of cellular energy [15]. Excessive use of fluoride may lead to dental fluorosis [16]. Therefore, safer alternatives such as maltitol (C_12_H_24_O_11_; 4-*O*-α-glucopyranosyl-D-sorbitol) could be used [17,18]. Maltitol is a hygroscopic non-reducing sugar and disaccharide sugar alcohol that is listed as an alternative sweetener; it has similar properties as sucrose and approximately 75–90% of the sweetness of sucrose [19].

In nature, maltitol is present in different fruits and vegetables, including roasted malt and chicory leaves. Commercially, maltitol is chemically synthesized from the starch of cereals such as corn, wheat and potatoes by the hydrogenation of maltose [17]. Due to the disadvantage of chemical process, green technology has been used as an alternative to improve the cost-effectiveness and low environmental impact. The biological processes have thus been developed for manufacturing steps. The present study focused on the novel biocatalytic process of transglycosylation reaction by CGTase for synthesizing maltitol products, having property in the prevention of tooth decay. The optimum condition for synthesizing maltitol products, identifying their structures, and their physiochemical, biological and antibacterial properties were investigated. In addition, this study aimed to gain a better understanding of the in vitro antibacterial activity of maltitol.

## 2. Materials and Methods

### 2.1. Materials

Maltooligosaccharide (G_1-4_), soluble potato starch, corn starch, bovine serum albumin and ampicillin sodium salt were purchased from Sigma-Aldrich (St. Louis, MO, USA). Tryptone and yeast extract were obtained from Difco (Bacton Dickinson and company, Sparks, MD, USA). β-CD was purchased from AppliChem (Darmstadt, Germany). Standard maltitol and D-sorbitol were obtained from TCI (Toshima, Kita-ku, Tokyo, Japan). Aquacide II, methanol, isopropanol, ethyl acetate, acetonitrile, HPLC-grade water, potassium dihydrogen phosphate, dipotassium phosphate and TLC silica gel 60 F254 glass plates (20 cm in height) were purchased from Merck (Darmstadt, Germany). IPTG, glycine and Tris-HCl were obtained from Vivantis (Sendirian Berhad, Shah Alam, Malaysia). Sulfuric acid was obtained from Carlo Erba Reagents (Val de Reuil, France). The commercial glucose oxidase kit was obtained from Human mit-diagnostics GmbH (Idstein, Germany). Other chemicals used were of an analytical grade.

### 2.2. Bacterial Cultivation and Enzyme Purification

The p19bBC recombinant plasmid was constructed by inserting the CGTase gene from *Bacillus circulans* A11 (GenBank accession No. AF302787) on a pET19b-based plasmid and transformed into *Escherichia coli* BL21 (DE3) [20,21]. The recombinant cells were grown in Luria–Bertani (LB) medium (pH 7.2) containing 100 μg/mL ampicillin at 37 °C for 24 h. The expression of recombinant CGTase was induced with 0.2 mM IPTG when the OD_600_ of the culture was estimated at 0.4–0.6. After 24 h, the cells were removed and the culture broth containing crude CGTase was collected by centrifugation at 18,000× *g* at 4 °C for 30 min. Subsequently, the crude CGTase was purified from the culture broth by starch adsorption and DEAE-Toyopearl 650M column chromatography, following the method of Charoensapyanan et al. [22]. The protein and CGTase-dextrinizing activity profiles of the eluted fractions were monitored by measuring absorbance at 280 nm and 600 nm, respectively [23,24]. Fractions containing CGTase-dextrinizing activity were pooled for further use, and the purity of the CGTase from each step in the purification process was determined using native-PAGE and SDS-PAGE [25].

### 2.3. Assay of CGTase

#### 2.3.1. Dextrinizing Activity

The dextrinizing activity of CGTase was determined by measuring the decrease in absorbance of a starch–iodine complex at 600 nm [23]. The CGTase reaction (50 μL) was incubated with 0.15 mL of 0.2% (*w*/*v*) soluble potato starch in 0.2 M phosphate buffer, pH 6.0, at 40 °C for 10 min. The reaction was terminated by the addition of 2 mL of 0.2 M HCl and 0.25 mL of iodine reagent (0.02% (*w*/*v*) I2) in 0.2% (*w*/*v*) KI). The mixture was adjusted to a final volume of 5 mL with distilled water, and the absorbance at 600 nm was measured. With the control, HCl was added before taking the CGTase. One unit of enzyme was defined as the amount of enzyme that produced a 10% reduction in the intensity of the blue color from the starch–iodine complex per min under the described conditions.

#### 2.3.2. Coupling Activity

The coupling activity of CGTase was measured by the glucose oxidase method [26]. The reaction mixture (100 μL) contained 40 μL of 1% (*w*/*v*) β-CD, 40 μL of 1% (*w*/*v*) cellobiose, 5 μL of the CGTase and 15 μL of 0.2 M phosphate buffer, pH 6.0. The reaction was performed at 50 °C for 5 min and halted by boiling for 5 min. Then, a 10 μL aliquot from the reaction mixture was added into 1 mL of glucose oxidase reagent. The glucose oxidase reaction was incubated at 37 °C for 5 min and then the absorbance at 500 nm was measured. The glucose concentration was calculated from the Equation (1).

Glucose concentration (μmol/mL) = 5.55 × A_Sample_/A_Std._ (1)

The absorbance of the standard solution (A_Std._) was measured by using 10 μL of a standard glucose (5.55 μmol/mL) with 1 mL of glucose oxidase reagent. One unit of coupling activity was defined as the amount of CGTase that produced 1 μmol of glucose per min under the assay conditions used.

### 2.4. Determination of Polyol-Acceptor Specificity of CGTase

In this study, the β-CD was selected as an appropriate donor, according to the result of Chaisin et al. [27] and Kaulpiboon et al. [28]. For the acceptor specificity of CGTase, various polyol acceptors were tested by intermolecular transglycosylation (coupling reactions). The 100 μL reaction mixtures of CGTase containing a final concentration of 1% (*w*/*v*) β-CD, 1% (*w*/*v*) different polyol acceptors (maltose, galactitol, mannitol, sorbitol, xylitol, arabitol, erythritol, maltitol) and CGTase (250 U/mL by dextrinizing activity) in 20 mM phosphate buffer, pH 6.0 were incubated at 50 °C for 24 h. The glucose produced from the CGTase transglycosylation reaction was measured using the glucose oxidase method.

### 2.5. Optimum Conditions for Maltitol Production

Before optimizing the conditions, the 250 U-CGTase reaction was incubated with its substrates at a final concentration of 1% (*w*/*v*) β-CD donor and sorbitol acceptor in 20 mM phosphate buffer at pH 6.0 and at 50 °C for 24 h. The optimum conditions for the synthesis of maltitol products were decided based on the highest yield (%) of synthetic maltitol, as determined from the TLC and HPLC results. The effects of varying the β-CD donor (0.5–2.5% (*w*/*v*)), sorbitol acceptor (0.5–2.5% (*w*/*v*)), CGTase unit (50–500 U/mL), incubation time (6–168 h), temperature (30–70 °C) and pH (3.0–11.0) were examined by a multifactor interaction test using a sequential approach.

### 2.6. Detection of Maltitol Product

#### 2.6.1. Thin Layer Chromatography (TLC) Analysis

The transfer products were analyzed on TLC silica gel 60 F_254_ at a height of 20 cm (Merck, Darmstadt, Germany). The mobile phase, composed of isopropanol:ethyl acetate:water (3:1:1, (*v*/*v*)), was used for running a TLC plate [27]. After running once, spots were detected by dipping with 1:9 (*v*/*v*) ratio of sulfuric acid:methanol followed by heating at 120 °C for 20 min. The intensity of the synthesized maltitol spots was quantified using a scanning densitometer (Model GS-800, Bio-Rad, Hercules, CA, USA). A commercial maltitol spot (50 μg) was used as the standard value.

#### 2.6.2. High-Performance Liquid Chromatography (HPLC) Analysis

The obtained products were also analyzed and purified by HPLC (Agilent Technologies 1260, Waldbronn, Germany) using a Luna^®^ 5 µm NH_2_ column (4.6 × 250 mm, Phenomenex, Torrance, CA, USA) and detected with a RI detector. The maltitol product mixture was filtered through a nylon membrane, 0.45 μm disc filter (Merck Millipore, Darmstadt, Germany) before injection and elution with acetonitrile:water (75:25, *v*/*v*) using a flow rate of 1.0 mL/min at 55 °C [29]. Product yields were determined using Equation (2), where t_0_ is the time the reaction commenced.

Product yield (%) = (peak area of product / peak area of acceptor at t_0_) × 100 (2)

### 2.7. Scale-Up Preparation and Purification of Synthesized Maltitol

The scaled-up production and purification of maltitol were performed under the optimized conditions described above. The transglycosylation volume of synthesis was increased to 100 mL. The synthesized maltitol was concentrated by evaporation and dialyzed with deionized water before injecting the 50 μL mixture into HPLC. The maltitol peak was separately collected and dried by lyophilization for further characterization.

### 2.8. Mass Spectrometry (MS) Analysis

The maltitol product was dissolved in 50% (*v*/*v*) methanol and put into the liquid chromatograph mass spectrometer (LCMS). The LCMS-ion trap-time-of-flight (LCMS-IT-TOF) profile was recorded on a microTOF (Shimadzu, Kyoto, Japan) at the Drug Discovery and Development Center and the Center of Scientific Equipment for Advanced Research, Thammasat University, Thailand. The compounds were ionized using electrospray ionization in the negative-ion mode. A flow of 1.5 L/min of nitrogen gas at 200 °C was established to nebulize the analytic solution to droplets using a nebulizer pressure set at 1 bar. A mass spectrometer was used to determine the molecular mass and purity of synthetic and commercial maltitols by separating ionic molecules according to their mass-to-charge ratio (*m*/*z*). The molecular mass and purity were calculated using Equations (3) and (4), respectively.

*m*/*z* = (molecular mass + number of protons)/charge (3)

MS sample purity (%) = (Height of single peak/Height of integrated peak) × 100(4)

### 2.9. NMR Analysis

The chemical structure of maltitol was identified by ^1^H and ^13^C-NMR spectra with Bruker’s AVANCE Tech HD 600 NMR spectrometer (Bruker, Billerica, MA, USA) at the Center of Scientific Equipment for Advanced Research, Thammasat University, Thailand. The operation took place at 600 MHz and at room temperature. The NMR spectra were obtained with 1 mg of sample dissolved in D_2_O.

### 2.10. Physiochemical and Biological Properties of Synthesized Maltitol

#### 2.10.1. Relative Sweetness

To compare the relative sweetness values of synthetic and commercial maltitol, 1% (*w*/*v*) solutions of both maltitols were measured using a refractometer (RX-1000, Atago Co., Ltd., Tokyo, Japan) and reported as °Brix values. Sucrose was used as the reference compound to plot the standard curve of the concentrations (%, *w*/*v*) versus the degrees Brix (°Bx) [30]. One degree Brix is defined as 1 g of sucrose in 100 mL solution.

#### 2.10.2. Growth-Inhibitory Effect of *Streptococcus mutans* DMST 18777 by Maltitol in Agar Media

From a stock culture, *S. mutans* was cultured at 37 °C for 48 h in brain heart infusion (BHI) agar under anaerobic conditions. A single colony of *S. mutans* from a freshly streaked agar plate was transferred to fresh BHI broth. Then, 1.5 × 10^8^ CFU/mL (0.5 Mcfarland standard) of precultured *S. mutans* were inoculated (1% (*v*/*v*)) in 50 mL of the BHI broth, the pH was adjusted to 7.4 and the bacterial cells were cultured for 18 h at 37 °C. The initial culture turbidity after inoculation was adjusted to an absorbance of 0.1 at a wavelength of 600 nm. For the spot assay on BHI agar [31], the BHI agar medium was supplemented with 2% (*w*/*v*) of glucose, sorbitol, commercial and synthesized maltitols; a control medium with no supplement was also used. All supplements of the BHI medium were filter sterilized using a 0.2 μm membrane (a syringe filter with a pore size of 0.2 μm; Merck Millipore, Burlington, MA, USA). *S. mutans* cells, obtained by centrifugation at 16,128× *g* for 10 min at 4 °C, were washed twice using 2 mM potassium phosphate buffer (PPB). The cells dilated by serial dilution: 10^3^-, 10^4^-, 10^5^-, 10^6^-, 10^7^- and 10^8^-fold using 2 mM PPB. An amount of 2 μL of each diluted cell suspension were spotted onto the BHI agar medium and cultured at 37 °C for 48 h.

#### 2.10.3. Antibacterial Activity

Disc Diffusion Technique

The disc diffusion technique was used to assess the susceptibility of *S. mutans* to each polyol. Filter paper discs (6 mm in diameter) were impregnated with a water solution containing 10, 20, 30 mg of sorbitol, and commercial and synthesized maltitol. The inoculum turbidity for each susceptibility test was adjusted to the 0.5 Mcfarland standard (1 × 10^8^ CFU/mL). The discs were placed on the surface of the *S. mutans* inoculated BHI agar. Ampicillin (25 μg/disc) was used as positive control, and sterile water was used as a negative control. The plates were then incubated under anaerobic conditions at 37 °C for 24 h. The inhibition zone was measured as the diameter surrounding the discs in millimeter. The experiments were performed in triplicate and the average value of inhibition zone was reported [22,32].

Minimal Inhibitory Concentration (MIC)

The MICs were determined in BHI broth by incubating *S. mutans* in the broth with variable amounts of the polyols and ampicillin. Briefly, the 0.5 Mcfarland culture (1 × 10^8^ CFU/mL) of *S. mutans* was diluted with BHI broth in a ratio of 1:10 (*v*/*v*). Two-fold serial dilutions (80 mg starter amount) of sorbitol, commercial maltitol and synthesized maltitol were prepared and 1 mL put in the test tubes. After 24 h of incubation at 37 °C under anaerobic conditions, the MICs were determined as the lowest concentrations of the ampicillin, sorbitol, commercial maltitol and synthesized maltitol, which inhibited the visible growth of *S. mutans*. MICs were measured in triplicate and the mean presented as mg/mL [32].

Minimal Bactericidal Concentration (MBC)

After the MIC tests were completed, the MBCs were examined by subculturing 200 μL from each test tube that showed no visible growth in the MIC test in BHI agar medium. After 24 h incubation at 37 °C under anaerobic condition, *S. mutans* growth on the BHI agar plates was investigated. The lowest concentration at which the ampicillin, sorbitol, standard maltitol and synthesized maltitol killed whole *S. mutans* represented the MBC. The MBC tests were performed in triplicate and the mean MBC was reported as mg/mL [22].

## 3. Results and Discussion

### 3.1. Purification and Acceptor Specificity of Recombinant CGTase

The purified recombinant CGTase from *B. circulans* A11 showed a specific activity of 1.40 × 10^4^ U/mg protein with a single protein band at molecular weight of 72 kDa; these values have been previously reported [22].

**Figure 1:**
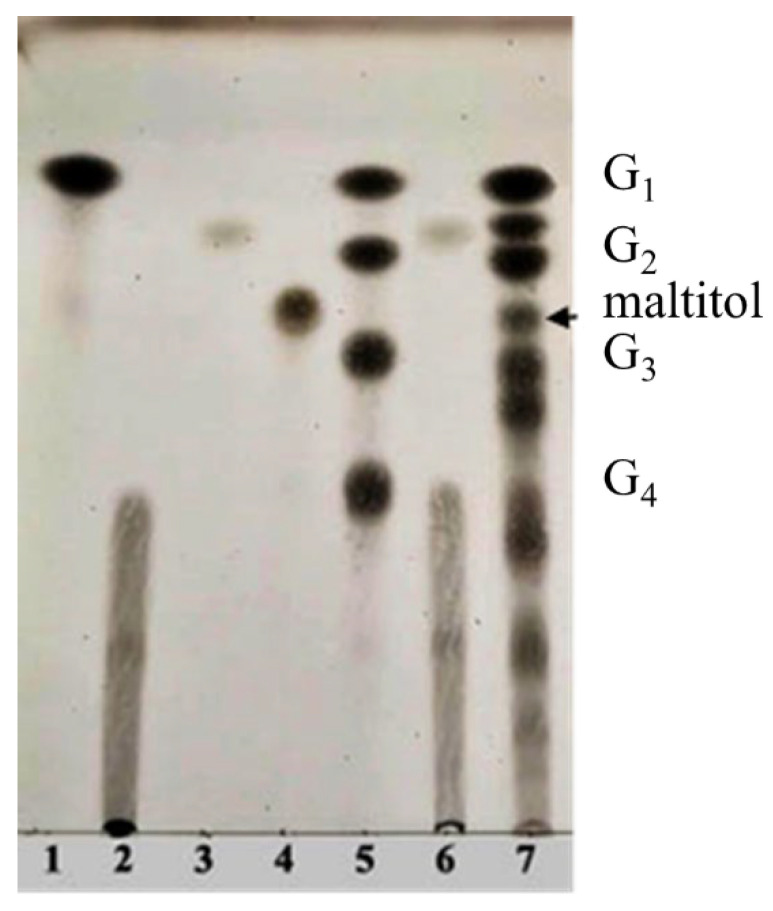
TLC analysis of the reaction products from recombinant CGTase incubated with 1% (*w*/*v*) of β-CD donor and sorbitol acceptor in 20 mM phosphate buffer, pH 6.0 at 50 °C for 24 h. Lane 1—standard glucose, 2—standard β-CD, 3—standard sorbitol, 4—standard maltitol, 5—standard G_1_, glucose; G_2_, maltose; G_3_, maltotriose and G_4_, maltotetraose, 6—control reaction without CGTase at 24 h, 7—reaction mixture with CGTase at 24 h.

The polyol-acceptor specificity of CGTase was determined from the transglycosylation activity assay using β-CD as a glucosyl donor as described in Section 2.4. The results showed that maltose had the highest activity, so it was set as 100% relative activity, followed by maltitol (C_12_, 4-*O*-α-glucopyranosyl-D-sorbitol), sorbitol (C_6_; six-carbon sugar alcohol), mannitol (C_6_), galactitol (C_6_), xylitol (C_5_), arabitol (C_5_), and erythritol (C_4_), at 48.7, 45.6, 44.2, 42.4, 41.6, 35.1 and 33.9% of activity, respectively (Table 1). These results suggested that CGTase preferred the disaccharide-polyol acceptor to monosaccharide-polyol acceptors, given the high coupling activity on maltose and maltitol. Principally, CGTase showed higher transglycosylation activity on the pyranose substrate, having one free hydroxyl group at the position C4-OH similar to glucose [33], maltose and maltitol. In the monosaccharide-polyol group, polyols with the six-carbon atoms were better acceptors for CGTase than five- and four-carbon atom polyols, and this explained the higher affinity for CGTase of sorbitol, a glucose-polyol chemically similar to glucose [34] compared to C_5_- and C_4_-sugar alcohols. The results also showed that the relative coupling activity of polyol isomer molecules such as sorbitol-mannitol and xylitol-arabitol isomers were almost equal. Few studies on polyol-acceptor specificity of CGTase have been reported; these have included D- and L-saccharide, alkyl glycoside, flavonoid and alcohol acceptors [22,27,35]. Kim et al. [7] reported the synthesis of glucosyl-sugar alcohols, mainly glucosyl-maltitol, using various transglycosylating enzymes including *B. macerans* CGTase (Amano Pharmaceutical Co., Ltd., Nagoya, Japan). They found that CGTase showed the highest transglycosylating activity on sugar alcohols compared to other enzymes (α-glucosidase, α-amylase and pullulanase), and maltitol (C_12_) and *myo*-inositol (C_6_) were the most effective polyol acceptors with transglycosylation yields of 49.9 and 46.5%, respectively. Later, Kim et al. [8] also synthesized transglucosylated xylitol using CGTase and studied its stimulating effect on the growth of *Bifidobacterium* sp. In this work, we focused on the synthesis of glucosyl-sorbitol (maltitol) and its anticariogenic role by inhibiting the cariogenic bacterium, *S. mutans*. Sorbitol had high CGTase activity, and there are no previously published data on the synthesis of glucosylsorbitol by CGTase. Therefore, sorbitol was chosen here to be a suitable glucosyl acceptor of this CGTase for our further study. To confirm that the obtained transglycosylation product consisted of maltitol, the reaction mixture under different conditions was run on TLC plate (Figure 1), included a control reaction without CGTase (Figure 1, Lane 6). After incubating for 24 h, the synthesized maltitol spot (*R*_f_ = 0.64) appeared at the same position as the standard maltitol which was between G_2_ (*R*_f_ = 0.72) and G_3_ (*R*_f_ = 0.60). In contrast, the control experiment did not show any product spots on TLC except β-CD and sorbitol. Thus, these results showed that the recombinant CGTase could transfer glucosyl residues from β-CD to sorbitol, yielding maltitol. In addition, it is possible that other transglycosylation and/or hydrolysis products were produced in the reaction mixture with sorbitol as the acceptor due to the multifunctional nature of CGTase (Figure 1, Lane 7).

### 3.2. Optimal Condition for the Production of Maltitol

**Figure 2:**
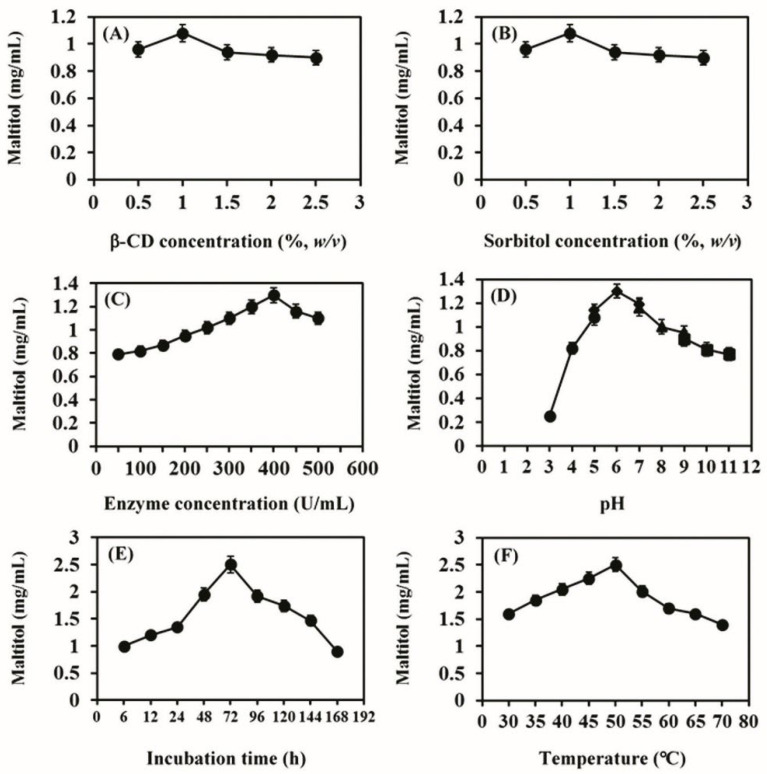
Maltitol production was affected by the following parameters: (**A**) β-CD concentration (donor substrate); (**B**) sorbitol concentration (acceptor substrate); (**C**) enzymatic concentration (U/mL); (**D**) pH-acetate (circle), phosphate (diamond), Tris-HCl (triangle) and glycine-NaOH (square) buffers; (**E**) incubation time and (**F**) temperature, where *w*/*v* is weight per volume and error bars indicate ± SD.

To produce the highest yield of maltitol, the parameters involved in the reaction were optimized by changing various conditions in the transglycosylation reaction, including the donor, acceptor and enzyme concentrations, pH, incubation time and temperature. The maltitol product that was produced from each reaction mixture was analyzed by TLC and quantified by comparison with the spot intensity of the standard maltitol. The optimal conditions for the production of maltitol were incubation of 1% (*w*/*v*) β-CD and 1% (*w*/*v*) sorbitol with 400 U/mL of CGTase in 20 mM phosphate buffer, pH 6.0, at 50 °C for 72 h (Figure 2). Under these conditions, the total yield of maltitol increased 2.5-fold (2.5 mg/mL or 25% yield) compared to the yield obtained before optimization (1.0 mg/mL or 10% yield). There have been few reports on the production of maltitol using sorbitol as an acceptor and bacterial enzymatic catalysis. Kim et al. [7] reported the synthesis of glucosyl-sorbitol (maltitol) from *B. macerans* CGTase with transglycosylation yield after 24 h of 15.6% (*w*/*w*); however, this product was not further characterized. Sato et al. [5] synthesized oligoglucosyl-inositol (56.6% (*w*/*w*) yield) from *myo*-inositol and β-CD; the transglycosylation reaction used *B. ohbensis* CGTase. Inositol was identified as a possible candidate as a prebiotic because it can stimulate the growth of *Bifidobacterium*. In previous studies, two glycosyl-polyols, glycosyl-maltitol (49.9% (*w*/*w*) yield) and transglycosylated xylitol (25% (*w*/*w*) yield, 7.8 g/L) were also synthesized with bacterial CGTase [7,36], and their structural characteristics and physiological functions were studied. Recently, Hu et al. [37] synthesized maltitol derivatives through an alternansucrase-catalyzed reaction with sucrose and maltitol substrates to obtain novel prebiotics. The reaction parameters were optimized and the obtained maltitol derivatives yields were 44.9% (*w*/*w*) (89.6 mg/mL), confirming the high ability of maltitol to act as an acceptor of alternansucrase. For commercial products, most of the maltitol was chemically produced through the hydrogenation of maltose and gave a high yield of 90% (*w*/*w*) [38]. Hence, the maltitol yield obtained in this study is quite good compared to the results of others under the biocatalytic process.

### 3.3. Upscale Production and HPLC Analysis

**Figure 3:**
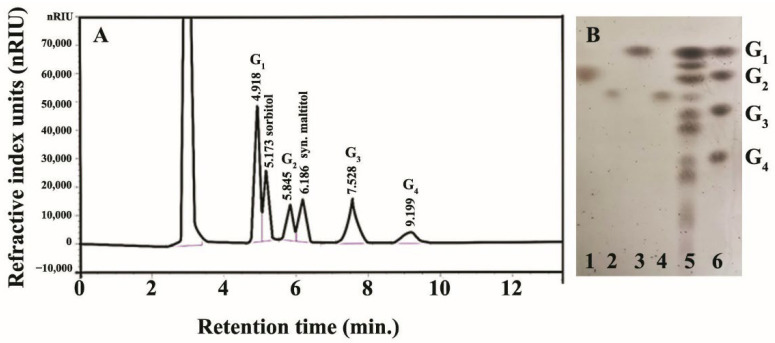
Detection of maltitol using (**A**) HPLC chromatogram of transglycosylation reaction catalyzed by recombinant CGTase and (**B**) TLC chromatogram from each HPLC fraction: Lane 1—at *R*_t_ 5.2 min (sorbitol), 2—1% (*w*/*v*) standard maltitol, 3—at *R*_t_ 4.9 min (G_1_), 4—at *R*_t_ 6.2 min (maltitol product), 5—the transglycosylation products of CGTase under optimal conditions as shown in Figure 2, 6—standard G_1_, glucose; G_2_, maltose; G_3_, maltotriose and G_4_, maltotetraose.

The reaction mixture for the production of maltitol from β-CD as a donor and sorbitol as an acceptor was scaled up to 100 mL, and the reaction proceeded under the optimum conditions according to Section 2.2. The maltitol product was then purified using HPLC, and separated at *R*_t_ of 6.2 min, while the residual substrate and other byproducts were observed at *R*_t_ of 5.2 (sorbitol), 4.9 (G_1_), 5.8 (G_2_), 7.5 (G_3_) and 9.2 (G_4_) min, respectively (Figure 3A). A large amount of reaction mixture was collected, pooled, lyophilized and rechecked for purity by TLC again (Figure 3B) before further structural analysis and bioactivity determination.

### 3.4. Mass Spectrometry (MS) Analysis

**Figure 4:**
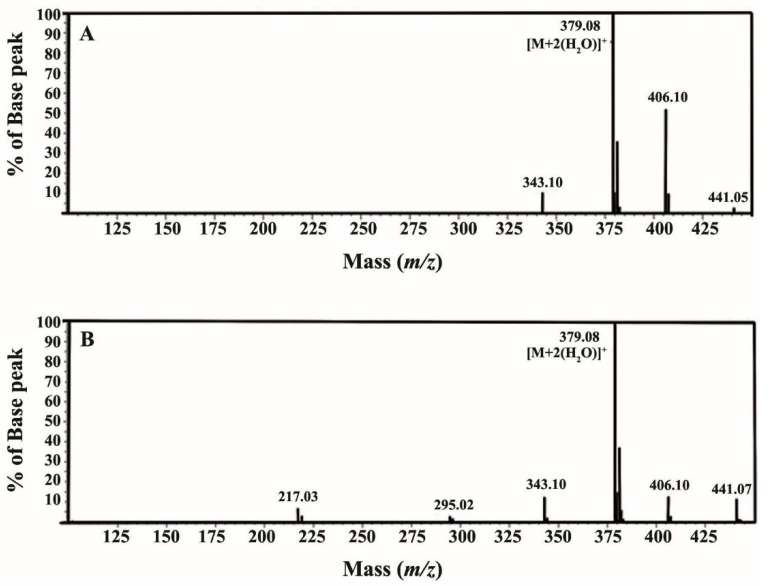
The LCMS-IT-TOF mass spectrum of (**A**) the synthetic and (**B**) commercial maltitols.

The molecular weight of synthesized maltitol product was elucidated by mass spectrometer in negative mode. The synthetic maltitol had a mass [M+2(H_2_O)]^+^ of 379.08 *m/z* (Figure 4) which was the same as standard maltitol, revealing [M+2(H_2_O)]^+^ at 379.08 *m/z*. This result confirms that our synthetic maltitol product was equivalent to standard maltitol (C_12_H_24_O_11_). In addition, purity of synthesized and commercial (std.) maltitols was also determined by LCMS and found to be ~62.29 and 68.96%, respectively.

### 3.5. NMR Analysis

**Figure 5:**
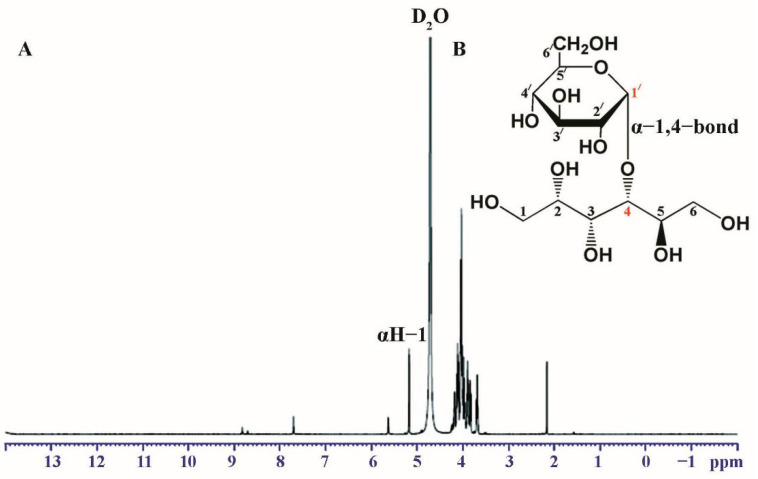
(**A**) The 600 MHz ^1^H-NMR spectrum and (**B**) the chemical structure of maltitol product.

The ^1^H- and ^13^C-NMR spectra of synthesized maltitol were determined. According to ^1^H-NMR (Figure 5A), the peak with doublet signals at 5.18 ppm (*J* = 3.91 Hz) was observed and assigned to the anomeric proton of glucose, which is bound to sorbitol via an α-1,4-glycosidic bond. In the ^13^C-NMR spectrum, the synthesized maltitol displayed 12 carbon signals (Table 2). Six carbon signals of each group (δ 63.81–74.18) and (δ 61.27–92.77) were assigned to sorbitol and glucose, respectively. After transglycosylation, the glucose residue was joined by an α-1,4-glycosidic bond between the α-anomeric form of C-1′ on the glucose and the hydroxyl oxygen atom on C-4 of the adjacent sorbitol. These results were confirmed by changing the chemical shift (δ) of the anomeric carbon of glucose (C-1′) from 92.77 to 100.42 ppm, while the δ of the C-4 position of sorbitol was deshielded from 72.61 to 82.05 ppm. Therefore, the combined NMR and MS analyses determined that the maltitol product is 4-*O*-α-glucopyranosyl-D-sorbitol (Figure 5B).

### 3.6. Characterization of the Synthesized Maltitol Product

#### 3.6.1. Relative Sweetness

The relative sweetness of synthesized maltitol was tested using sucrose as the reference by measuring the Brix level with a refractometer. The sweetness value of synthetic maltitol was 0.9, which was equal to standard maltitol [17].

#### 3.6.2. Growth Inhibitory Effect of the Synthesized Maltitol on *Streptococcus mutans* DMST 18777

**Figure 6:**
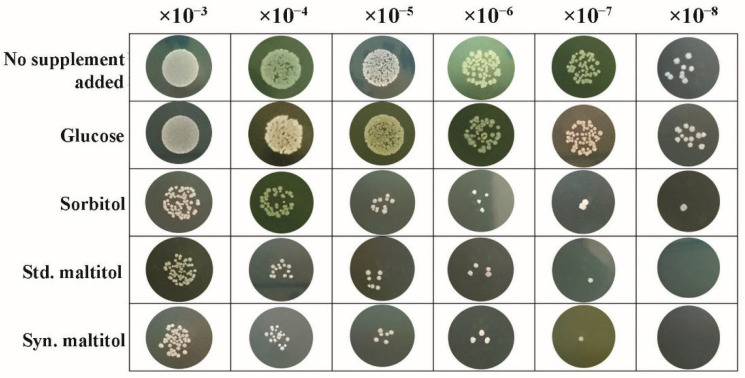
A spot assay was performed by cultivating *S. mutans* DMST 18777 on a BHI solid medium supplemented with 2% (*w*/*v*) of no sugar, glucose, sorbitol, std. maltitol or syn. maltitol as a carbon source at 37 °C for 48 h.

The results of the spot assay indicated that the numbers of *S. mutans* colonies were lower in the presence of standard and synthetic maltitols compared to no additional supplement, glucose and sorbitol (Figure 6). In the BHI agar medium containing 2% (*w*/*v*) of standard and synthetic maltitols, *S. mutans* colonies did not form when the plates were seeded with bacterial inoculate at a 10^8^ dilution. In contrast, *S. mutans* colonies formed on the agar plate when inoculated with bacterial dilutions of either 10^7^ or 10^8^, with supplement, glucose and sorbitol. Therefore, 2% (*w*/*v*) of standard and synthetic maltitols had antibacterial activity. For other polyols, in vivo and in vitro antibacterial activity has been identified with xylitol and erythritol against cariogenic bacteria, including *Streptococcus mutans* and *Streptococcus sobrinus* [39,40,41]. The mechanism was assumed to be polyol accumulation in the bacteria, resulting in the formation of a toxic sugar phosphate [42]. Keijser et al. [18] reported that the daily use of maltitol-sweetened gum on dental plaques reduced the quantity of several bacterial species in 153 volunteers, notably, *Actinomyces* species, which play an important role in the early formation of dental biofilms. They found that maltitol exposure led to low expression of the phosphoenolpyruvate-dependent sugar phosphotransferase system (PEP-PTS system), that were possibly related to low growth rates. With the spot assay, although maltitol gave the same result in the growth inhibition of *S. mutans* as sorbitol, it was safer for the user than sorbitol. The maltitol has less adverse effects, is sweeter and has less calories than sorbitol. Moreover, in normal people, the conversion of sorbitol to fructose by sorbitol dehydrogenase is critical because it allows any excess sorbitol to be excreted from the cell, and sorbitol cannot exit the cell once it is formed. If sorbitol levels continue to increase within cells, they create an osmotic effect. Intracellular accumulation of sorbitol is a major factor in the development of most complications of diabetes [43].

#### 3.6.3. Antibacterial Activity

By disc diffusion (Table 3), standard and synthesized maltitols (40 mg/disc) exhibited moderate antibacterial activity against *S. mutans*, with inhibition zones of 14.0 ± 3.0 mm. The lowest MIC and MBC values for synthesized maltitol were 20 and 40 mg/mL against *S. mutans*, respectively (Table 4). Additionally, synthesized maltitol had greater antibacterial activity than sorbitol and similar activity as standard maltitol. To understand the possible inhibitory mechanism of polyols on *S. mutans*, Yun et al. [31] found that xylitol polyol had an inhibitory effect on the glucose uptake of *S. mutans* cells. Moreover, xylitol is transported into *S. mutans* following phosphorylation of xylitol to xylulose-5-phosphate (X5P) by the PEP-PTS system, and that X5P accumulates in *S. mutans*, inhibiting enzymes in the glycolysis pathway such as glucose-6-phosphate isomerase and phosphofructokinase [31]. Another mechanism for the inhibitory effect of maltitol on *S. mutans* might be its inability to be fermented by *S. mutans*, similar to xylitol, resulting in a lack of cellular energy [8,37].

## 4. Conclusions

Recombinant p19bBC CGTase from *Bacillus circulans* A11 showed effective transglycosylation activity with sorbitol, yielding maltitol with maltooligosaccharide byproducts. Under optimal conditions, the maximum yield of synthetic maltitol obtained was 25% (*w*/*v*) or 2.5 mg/mL. The chemical structure of synthetic maltitol was confirmed by LCMS-IT-TOF and NMR analyses to be 4-*O*-α-glucopyranosyl-D-sorbitol. Maltitol was effective at inhibiting the cariogenic *S. mutans* and could be used in various food and dental care products such as anticariogenic sugar, chewing gum and toothpaste to counter tooth decay. In future research, we are interested in synthesizing long-chain maltitol using CGTase and apply it as a prebiotic.

## Figures and Tables

**Table 1:** Acceptor specificity of recombinant CGTase and the structure of the different substrates tested.

Acceptor	C-Atom	Chemical Formula	Synonym	Chemical Structure	Relative Activity ^a^ (%)
Maltitol	12	C_12_H_24_O_11_	4-*O*-α-Glucopyranosyl-D-sorbitol	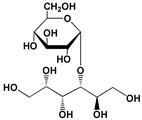	48.7 ± 5.3
Sorbitol	6	C_6_H_14_O_6_	D-Glucitol, D-Sorbitol	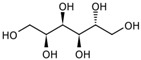	45.6 ± 3.7
Mannitol	6	C_6_H_14_O_6_	Mannite, D-Mannitol	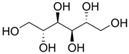	44.2 ± 2.4
Galactitol	6	C_6_H_14_O_6_	Dulcitol, Dulcite	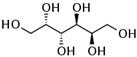	42.4 ± 2.3
Xylitol	5	C_5_H_12_O_5_	Xylite	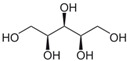	41.6 ± 2.4
Arabitol	5	C_5_H_12_O_5_	D-(+)-Arabitol	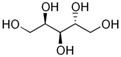	35.1 ± 1.9
Erythritol	4	C_4_H_10_O_4_	meso-Erythritol, meso-1,2,3,4-Tetrahydroxybutane, 1,2,3,4-Butanetetrol, i-Erythritol	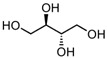	33.9 ± 2.0

^a^ Relative coupling activity of CGTase was performed with 1% (*w*/*v*) of β-CD and maltose or different polyols in 20 mM phosphate buffer, pH 6.0 at 50 °C for 24 h. Data are shown as mean ± SD and derived from three independent repeats.

**Table 2:** ^13^C-NMR Data of synthesized maltitol product.

Carbon Atom		^13^C-NMR (δ ^a^, ppm)		
		Sorbitol	Glucose	Maltitol
C-sorbitol	1	63.81		63.26
	2	74.18		74.30
	3	71.09		72.10
	4	72.61		82.05
	5	72.53		72.56
	6	64.23		62.53
C-glucose	1′		92.77	100.42
	2′		72.15	71.89
	3′		73.43	72.86
	4′		70.32	70.27
	5′		72.10	71.98
	6′		61.27	61.08

^a^ Values of chemical shift (δ) are stated in ppm.

**Table 3:** Antibacterial activity of ampicillin, sorbitol and the standard and synthesized maltitols against *Streptococcus mutans* DMST 18777.

*Streptococcus mutans* DMST 18777	Total Amount	Zone of Inhibition in mm of Diameter ^a^
Ampicillin	10 μg	24.0 ± 2.0
Sorbitol	10 mg	0.0
	20 mg	0.0
	30 mg	0.0
	40 mg	8.0 ± 2.0
Std. maltitol	10 mg	0.0
	20 mg	7.2 ± 2.0
	30 mg	9.0 ± 2.0
	40 mg	14.0 ± 2.0
Syn. maltitol	10 mg	0.0
	20 mg	7.9 ± 2.0
	30 mg	10.0 ± 1.0
	40 mg	14.0 ± 3.0

^a^ Values of inhibitory zone in mm are the mean ± SD of three parallel measurements.

**Table 4:** The minimal inhibitory concentration (MIC) and minimal bactericidal concentration (MBC) of ampicillin, sorbitol and the standard and synthesized maltitols against *Streptococcus mutans* DMST 18777.

*S. mutans* DMST 18777	Ampicillin (µg/mL)	Sorbitol (mg/mL)	Standard Maltitol (mg/mL)	Synthesized Maltitol (mg/mL)
MIC ^a^	0.156 ± 0	40 ± 0	20 ± 0	20 ± 0
MBC ^a^	0.625 ± 0	80 ± 0	40 ± 0	40 ± 0

^a^ All data are shown as mean ± SD derived from triplicate experiments.

## Data Availability

Data are contained within the article.
